# Supplementary material for: From sequence to enzyme mechanism using multi-label machine learning
Source: BMC Bioinformatics. 2014 May 19;15:150. doi: 10.1186/1471-2105-15-150 (PMC4229970; doi:10.1186/1471-2105-15-150)
Supplement: Additional file 2 — Java code of ml2db. Additional file ml2db_code.tar.gz contains the Java source code to run the multi-label machine learning experiments and save the results to database. The code’s Javadoc is included. [file 1471-2105-15-150-S2.zip › additional file 2/ml2db/ml2db/doc/mulan/evaluation/MultipleEvaluation.html]

MultipleEvaluation


JavaScript is disabled on your browser.


- Overview
- Package
- Class
- Use
- Tree
- Deprecated
- Index
- Help

- Prev Class
- Next Class

- Frames
- No Frames

- All Classes

- Summary:
- Nested |
- Field |
- Constr |
- Method

- Detail:
- Field |
- Constr |
- Method


mulan.evaluation

## Class MultipleEvaluation

- java.lang.Object
- - mulan.evaluation.MultipleEvaluation

- ---

    

  ```
  public class MultipleEvaluation
  extends java.lang.Object
  ```

  Simple class that includes an array, whose elements are lists of evaluation
  evaluations. Used to compute means and standard deviations of multiple
  evaluations (e.g. cross-validation)

  Author:
  :   Grigorios Tsoumakas

- - ### Constructor Summary

    Constructors

    Constructor and Description

    `MultipleEvaluation(Evaluation[] someEvaluations,
    MultiLabelInstances data)`

    Constructs a new object with given array of evaluations

    `MultipleEvaluation(MultiLabelInstances data)`

    Constructs a new object
  - ### Method Summary

    Methods

    | Modifier and Type | Method and Description |
    | --- | --- |
    | `void` | `addEvaluation(Evaluation evaluation)` Adds an evaluation results to the list of evaluations |
    | `void` | `calculateStatistics()` Computes mean and standard deviation of all evaluation measures |
    | `double` | `getMean(java.lang.String measureName)` Returns the mean value of a measure |
    | `java.lang.String` | `toCSV()` Returns a CSV string representation of the results |
    | `java.lang.String` | `toString()` Returns a string with the results of the evaluation |

    - ### Methods inherited from class java.lang.Object

      `equals, getClass, hashCode, notify, notifyAll, wait, wait, wait`

- - ### Constructor Detail


    - #### MultipleEvaluation

      ```
      public MultipleEvaluation(MultiLabelInstances data)
      ```

      Constructs a new object

      Parameters:
      :   `data` - the evaluation data used for obtaining label names for per
          outputting per label values of macro average measures


    - #### MultipleEvaluation

      ```
      public MultipleEvaluation(Evaluation[] someEvaluations,
                        MultiLabelInstances data)
      ```

      Constructs a new object with given array of evaluations

      Parameters:
      :   `data` - the evaluation data used for obtaining label names for per
          outputting per label values of macro average measures
      :   `someEvaluations` -
  - ### Method Detail


    - #### calculateStatistics

      ```
      public void calculateStatistics()
      ```

      Computes mean and standard deviation of all evaluation measures


    - #### addEvaluation

      ```
      public void addEvaluation(Evaluation evaluation)
      ```

      Adds an evaluation results to the list of evaluations

      Parameters:
      :   `evaluation` - an evaluation result


    - #### toString

      ```
      public java.lang.String toString()
      ```

      Returns a string with the results of the evaluation

      **Overrides:**
      :   `toString` in class `java.lang.Object`

      Returns:
      :   a string with the results of the evaluation


    - #### getMean

      ```
      public double getMean(java.lang.String measureName)
      ```

      Returns the mean value of a measure

      Parameters:
      :   `measureName` - the name of the measure

      Returns:
      :   the mean value of the measure


    - #### toCSV

      ```
      public java.lang.String toCSV()
      ```

      Returns a CSV string representation of the results

      Returns:
      :   a CSV string representation of the results


- Overview
- Package
- Class
- Use
- Tree
- Deprecated
- Index
- Help

- Prev Class
- Next Class

- Frames
- No Frames

- All Classes

- Summary:
- Nested |
- Field |
- Constr |
- Method

- Detail:
- Field |
- Constr |
- Method
